# Contribution of nuclear BCL10 expression to tumor progression and poor prognosis of advanced and/or metastatic pancreatic ductal adenocarcinoma by activating NF-κB-related signaling

**DOI:** 10.1186/s12935-021-02143-z

**Published:** 2021-08-19

**Authors:** Sung-Hsin Kuo, Shih-Hung Yang, Ming-Feng Wei, Hsiao-Wei Lee, Yu-Wen Tien, Ann-Lii Cheng, Kun-Huei Yeh

**Affiliations:** 1 Department of Oncology, National Taiwan University Hospital and National Taiwan University College of Medicine, No. 7, Chung-Shan S Rd, Taipei, Taiwan; 2 Department of Surgery, National Taiwan University Hospital and National Taiwan University College of Medicine, Taipei, Taiwan; 3 Department of Internal Medicine, National Taiwan University Hospital and National Taiwan University College of Medicine, Taipei, Taiwan; 4 Cancer Research Center, National Taiwan University College of Medicine, Taipei, Taiwan; 5 Graduate Institute of Oncology, National Taiwan University College of Medicine, Taipei, Taiwan; 6 Graduate Institute of Clinical Medicine, National Taiwan University College of Medicine, Taipei, Taiwan; 7 Department of Oncology, National Taiwan University Cancer Center, National Taiwan University College of Medicine, Taipei, Taiwan

**Keywords:** Pancreatic cancer, BCL10, NF-κB, Prognosis

## Abstract

**Background:**

We previously demonstrated that nuclear BCL10 translocation participates in the instigation of NF-κB in breast cancer and lymphoma cell lines. In this study, we assessed whether nuclear BCL10 translocation is clinically significant in advanced and metastatic pancreatic ductal adenocarcinoma (PDAC).

**Method and materials:**

We analyzed the expression of BCL10-, cell cycle-, and NF-κB- related signaling molecules, and the DNA-binding activity of NF-κB in three PDAC cell lines (mutant *KRAS* lines: PANC-1 and AsPC-1; wild-type *KRAS* line: BxPC-3) using BCL10 short hairpin RNA (shBCL10). To assess the anti-tumor effect of BCL10 knockdown in PDAC xenograft model, PANC-1 cells treated with or without shBCL10 transfection were inoculated into the flanks of mice. We assessed the expression patterns of BCL10 and NF-κB in tumor cells in 136 patients with recurrent, advanced, and metastatic PDAC using immunohistochemical staining.

**Results:**

We revealed that shBCL10 transfection caused cytoplasmic translocation of BCL10 from the nuclei, inhibited cell viability, and enhanced the cytotoxicities of gemcitabine and oxaliplatin in three PDAC cell lines. Inhibition of BCL10 differentially blocked cell cycle progression in PDAC cell lines. Arrest at G1 phase was noted in wild-type *KRAS* cell lines; and arrest at G2/M phase was noted in mutant *KRAS* cell lines. Furthermore, shBCL10 transfection downregulated the expression of phospho-CDC2, phospho-CDC25C, Cyclin B1 (PANC-1), Cyclins A, D1, and E, CDK2, and CDK4 (BxPC-3), p-IκBα, nuclear expression of BCL10, BCL3, and NF-κB (p65), and attenuated the NF-κB pathway activation and its downstream molecule, c-Myc, while inhibition of BCL10 upregulated expression of p21, and p27 in both PANC-1 and BxPC-3 cells. In a PANC-1-xenograft mouse model, inhibition of BCL10 expression also attenuated the tumor growth of PDAC. In clinical samples, nuclear BCL10 expression was closely associated with nuclear NF-κB expression (p < 0.001), and patients with nuclear BCL10 expression had the worse median overall survival than those without nuclear BCL10 expression (6.90 months versus 9.53 months, p = 0.019).

**Conclusion:**

Nuclear BCL10 translocation activates NF-κB signaling and contributes to tumor progression and poor prognosis of advanced/metastatic PDAC.

**Supplementary Information:**

The online version contains supplementary material available at 10.1186/s12935-021-02143-z.

## Introduction

The majority of patients with pancreatic ductal adenocarcinoma (PDAC) are diagnosed when the cancer becomes un-resectable and metastatic [1, 2]. The median survival for patients with locally advanced PDAC, who received combined chemotherapy and radiotherapy or chemotherapy alone, varies from 7 to 21 months [3, 4]. However, the median survival for patients with metastatic PDAC is approximately 3–6 months after treatment with single-agent or combinational chemotherapy, or with targeted therapy [4, 5]. In contrast to the efficacies of epidermal growth factor receptor- or vascular endothelial growth factor-targeting agents in many solid tumors, aforementioned molecular targeted agents exhibit limited therapeutic efficacies in patients with PDAC [6–8].

Tumor microenvironments, for example, pancreatic stellate cells (main components of the stroma) can promote the proliferation, invasion, metastasis, and drug resistance of pancreatic cancer cells [9, 10]. Previous studies have demonstrated that sonic hedgehog (Shh) ligands facilitate cell proliferation of PDAC through triggering nuclear factor (NF)-κB [11, 12]. Our previous work showed that nuclear expression of NF-κB or Gli1 (a determinant of Shh signaling) was associated with poor overall survival (OS) of patients with advanced and metastatic PDAC after commencing chemotherapy [13]. These findings suggested that proinflammatory cytokines or growth factors can activate NF-κB signaling in stromal or cancer cells of PDAC though autocrine or paracrine loop.

BCL10 (B-cell CLL/lymphoma 10), cloned from the t(1;14)(p22;q32), is involved in the pathogenesis of mucosa-associated lymphoid tissue (MALT) lymphoma [14, 15]. When BCL10 is expressed in the cytoplasm of normal T and B cells, it communicates antigen receptor mediated signals and further activates NF-κB signaling [16, 17]. We have previously reported that nuclear translocation and co-localization of BCL10 and NF-κB are closely associated with antibiotic-unresponsiveness of gastric MALT lymphoma [18, 19]. In human breast cancer cells (MCF7 cell lines), we revealed that AKT, activated by tumor necrosis factor-alpha (TNF-α), phosphorylates BCL10 at Ser^218^ and Ser^231^, which subsequently forms a complex with BCL3 (containing a nuclear localization signal) to enter the nucleus and further activate NF-κB signaling [20]. Furthermore, we reported that BCL10 regulates tumor cell growth of cervical cancer cells by regulating the activation of NF-κB-dependent Cyclin D1 signaling [21]. These results revealed an association between nuclear BCL10 translocation and the activation of NF-κB in certain subtypes of solid cancers. However, little is known about the biological characteristics of BCL10 in PDAC, although the expression of BCL10 has previously been reported in pancreatic acinar cell cancer [22].

Considering that NF-κB activation has been shown to be associated with the pathogenesis and prognosis of PDAC [13, 23, 24], we sought to investigate whether BCL10 signaling and its association with NF-κB activation possesses any clinical significance with respect to PDAC. We first assessed the BCL10-mediated regulation of cell proliferation and activation of NF-κB in PDAC through both in vitro and in vivo models. Subsequently, we examined the association between nuclear BCL10 expression patterns in tumor cells and the clinical outcomes of patients with recurrent, advanced, and metastatic PDAC who received systemic chemotherapy.

## Methods and materials

### Pancreatic cancer cell lines

Although the point mutations of *KRAS* on codon 12 (exon 2) consist of major initiating oncogenic events and progression processes in approximately 80% of patients with pancreatic ductal adenocarcinoma (PDAC) [25, 26], *KRAS* mutations were not detected in some patients with PDAC. In East Asian patients with PDAC, including Taiwanese patients, the frequencies of *KRAS* mutations in codon 12 (exon 2) have been reported to range from 72 to 90% [27–29]. In other words, approximately 15–20% of patients harbored wild-type *KRAS* PDAC. Previous evidence has demonstrated that *KRAS* mutation can activate the KRAS protein, which further triggers a cascade of survival signaling, including phosphoinositide 3-kinase (PI3K)/AKT/mechanistic target of rapamycin (mTOR), p38 mitogen-activated protein kinase (MAPK), extracellular signal- regulated kinase (ERK), c-Jun N-terminal kinase (JNK), and NF-κB, and thus promotes proliferation, migration, invasiveness, and causes further progression and poor prognosis of PDAC [30, 31]. However, the underlying molecular mechanisms contributing to tumor proliferation, migration, invasiveness, and progression in patients with wild-type *KRAS* PDAC remain unclear. To investigate whether BCL10 is involved in the molecular pathogenesis, treatment efficacy, and prognosis of patients with advanced and metastatic PDAC irrespective of *KRAS* mutation status, we selected three PDAC cell lines, including mutant *KRAS* cell lines (PANC-1 and AsPC-1) and wild-type cell line (BxPC-3) in this study, to mimic the in vitro biology of PDAC and further explore the underlying molecular mechanisms regulated by nuclear BCL10.

The human PDAC cell lines, PANC-1 (mutant *KRAS*), AsPC-1 (mutant *KRAS*), and BxPC-3 (wild type *KRAS*), were purchased from American Type Culture Collection (ATCC). The PANC-1 cell line was maintained in complete Dulbecco’s modified Eagle’s medium (DMEM), whereas AsPC-1 and BxPC-3 were maintained in complete Roswell Park Memorial Institute (RPMI)-1640. Furthermore, all cell lines were cultured in an incubator containing a humidified atmosphere and 5% CO_2_ at 37 °C.

### The shRNA-mediated BCL10 silencing in PDAC cell lines

Previously, we demonstrated that TNF-α expression resulted in the BCL10 nuclear translocation due to phosphorylation of BCL10 Ser^218^ or Ser^231^ in MCF7 cells [20]. The short hairpin RNA (shRNA)-expression lentiviral vector of BCL10 (TRCN0000359256) was obtained from the National RNAi Core Facility (Taipei, Taiwan), and the silenced site was located in the Ser^231^ of BCL10 (Additional file 1: Figure S1). For construction of the lentiviruses, BCL10 shRNA transfected (shBCL10) vectors, psPAX2 (Addgene), and pMD2.G (Addgene), were cotransfected into 293 T cells. At 48 h after transfection, the supernatant was collected and used to infect the three PDAC cell lines. The pGIPZ lentiviral vector (Fisher Scientific) was used as a scrambled control.

### Cell proliferation and cytotoxicity assay

The PDAC cells with or without transfection of shBCL10 lentivirus were seeded into 96-well plates at a density of 1000 cells/well. Every 2 days, the levels of cell proliferation were assessed using a Premixed WST-1 Cell Proliferation Reagent (Clontech). The absorbance was detected at 450 nm against the background control using a multi-well plate reader. The cell proliferation rate was calculated as follows: Proliferation rate = (absorbance/absorbance of Day 1) × 100%. The results are revealed as the mean ± standard error (SE) of three different experiments.

For cytotoxicity assay, cells were seeded into 96-well plates at a density of 3000 cells/well, and then, treated with gemcitabine and oxaliplatin at various doses. After incubation at 37 °C for 72 h, cell viability was also measured using Premixed WST-1 Cell Proliferation Reagent. The data are expressed as the mean ± SE of three different experiments. *p < 0.05; **p < 0.01; ***p < 0.001.

### Flow cytometric analysis

The untransfected, scramble shRNA (Scr) transfected, and shBCL10 transfected PDAC cells seeded into 6-cm dishes were detached using 0.05% trypsin–EDTA (Gibco). The harvested cells were washed twice with phosphate buffered saline (PBS), and then, fixed in 70% cold ethanol at 4 °C overnight. Fixed cells were washed twice with PBS, incubated with 50 µg/ml RNase at 37 °C for 30 min, and stained with propidium iodide (PI) (50 µg/mL) on ice for 30 min. The cell cycle phases of stained cells were analyzed in the FL-2 channel using a FACSCalibur (Becton Dickinson, BD FACSCalibur™ system).

### Confocal immunofluorescence

The shBCL10 untreated and treated PDAC cells were plated on cell culture slide (SPL Life Sciences) overnight. After washing twice with PBS, the cells fixed with 3.7% paraformaldehyde at 4 °C for 8 min, permeabilized with 0.1% TritonX-100 in PBS for 10 min, and blocked with 3% BSA at room temperature for 1 h. Then, cells were incubated with the anti-BCL10 primary antibody (1:200) (sc-9560; Santa Cruz Biotechnology, Santa Cruz, CA, USA) overnight. Subsequently, the cells were washed with PBS and incubated with DyLight 488 conjugated secondary antibody (1:1000) (Thermo Fisher Scientific) for 1 h. Nuclei were counterstained with DAPI (dilution 1:1000 in PBS) (Biotium, 40043) for 5 min. The stained cells were examined using a confocal laser scanning microscope (TCS SP5, Leica Microsystems) and a Leica Application Suite 2.02.

### Immunoblotting analysis

Whole cell lysates and nuclear lysates of PADC cells were harvested from each cell subclones. Equal amounts of protein extracts were fractionated on sodium dodecyl sulfate (SDS)-Tris glycine polyacrylamide gel electrophoresis (PAGE) gel and then electrophoretically transferred to polyvinylidene difluoride (PVDF) membrane (Millipore). Primary antibodies against the following molecules were used for the analysis: BCL10 (sc-9560; Santa Cruz Biotechnology, Santa Cruz, CA, USA), BCL3 (sc-185; Santa Cruz Biotechnology), phospho (p)-IκBα (Thr^389^; #2905; Cell Signaling Technology), NF-κB (p65; #9451; Santa Cruz Biotechnology), p-p65 (Ser^536^, 93H1; Cell Signaling Technology), c-Myc (sc-40; Santa Cruz Biotechnology), cyclin-dependent kinases (CDK)2 (#18048; Cell Signaling Technology), CDK4 (#12790; Cell Signaling Technology), cell division cycle (CDC)2 (#28439; Cell Signaling Technology), p-CDC2 (#4539; Cell Signaling Technology), CDC25C (#4688; Cell Signaling Technology), p-CDC25C (#4901; Cell Signaling Technology), Cyclin A (#53230; Santa Cruz Biotechnology), Cyclin B1 (sc-752; Santa Cruz Biotechnology), Cyclin D1 (#2972; Cell Signaling Technology), Cyclin E (sc-9566; Santa Cruz Biotechnology), p21: (#2947; Cell Signaling Technology), p27 (#SC-528; Santa Cruz Biotechnology), β-actin (A5316, Sigma-Aldrich, MO, USA), α-tubulin (CP06, Calbiochem, San Diego, CA, USA), and GAPDH (#5174; Cell Signaling Technology) [19, 21, 32–34]. The specific reactive bands on membranes were probed using appropriate secondary IgG antibodies conjugated to horse radish peroxidase. The immune complexes were visualized using an enhanced chemiluminescence detection system (ECL, Boehringer Mannheim, Mannheim) and quantification was performed using the Image Quant software (GE Healthcare). All experiments were repeated at least three times.

### Luciferase assays

For the NF-κB promoter analysis, PANC-1 and BxPC-3 cells were transfected with 5 µg of NF-κB-Luc reporter plasmid DNA (BD Bioscience, Clontech, Palo Alto, CA, USA) for 6 h by using Transfast Transfection Reagent (Promega, Madison, WI, USA). The luciferase activity of the cell lysates was measured according to the luciferase assay kit manual (Promega) and normalized for the amount of protein in each cell lysate [19, 21].

### In vivo animal experiments

Male NOD.CB17-Prkdc^scid^ mice were purchased from the National Laboratory Animal Center, Taiwan. Our animal experimental procedures were approved by the National Taiwan University College of Medicine and College of Public Health Institutional Animal Care and Use Committee (IACUC; Number 20150056). Approximately 1 × 10^6^ scrambled PANC-1 cells and shBCL10-transfected PANC-1 cells were injected subcutaneously into the flanks of mice; and all mice received anesthesia with isoflurane/oxygen (2.5% isoflurane) for 5 min during injection of PANC-1 cells. Body weight and tumor volumes of mice were observed after implantation and measured once a week. Tumor volume was measured with calipers and calculated using the equation: 0.5 × L × W^2^, where L is the length and W is the width. At the endpoint of animal experiment, all mice were euthanized with asphyxiation using carbon dioxide (CO_2_) which introduced into the chamber at the rate of 30% replacement of chamber air per minute.

### Patients, tumor samples, and immunohistochemical analyses

In order to reduce underlying drug resistance of different chemotherapies of bios, and the different chemotherapy regimen-associated responses and subsequent survival, we selected patients who received gemcitabine-based chemotherapy as first-line treatment after initial diagnosis of locally advanced and metastatic PDAC or at the status of recurrent and/or distant metastatic PDAC after curative surgery. Patients who were diagnosed only by cytological examination were excluded. We reviewed the medical records and histopathological data of patients with PDAC who met the aforementioned criteria. This cohort included adequate amounts of archival tissues, including biopsy or surgery (recurrent cases) for immunohistochemical evaluation, complete medical records documenting the disease course, and the histological diagnosis of PDAC. The study was approved by the Research Ethical Committee of National Taiwan University Hospital (Institutional. Review Board Number: 201507023RINA).

Formalin-fixed, paraffin-embedded tumor tissue sections (4 μm thick) were immunohistochemically stained using an avidin–biotin-peroxidase method as described previously [13]. The primary antibodies and their dilutions were as follows: anti-BCL10 (sc-9560; Santa Cruz Biotechnology) and anti-NF-κB p65 (sc-109, Santa Cruz Biotechnology). BCL10 and NF-κB nuclear expression were considered positive when more than 10% of tumor cells exhibited positively stained nuclei [13, 18].

### Statistical analysis

The data from the WST-1 assays and cell cycle analysis are expressed as the mean ± SE of experiments repeated at least three times. Statistical analysis was determined using Student’s *t* test, Χ^2^ test, and Fisher’s exact test. The OS was measured from the date of the commencement of chemotherapy to the date of death from any cause or the last follow-up date. Univariate analysis was performed using Kaplan–Meier survival curves (log-rank test) to evaluate the statistical significance of OS differences among patients with tumors either or not expressing nuclear BCL10. A p value of < 0.05 was considered statistically significant.

## Results

### Downregulation of BCL10 attenuates the proliferation of PDAC cells

**Fig. 1:**
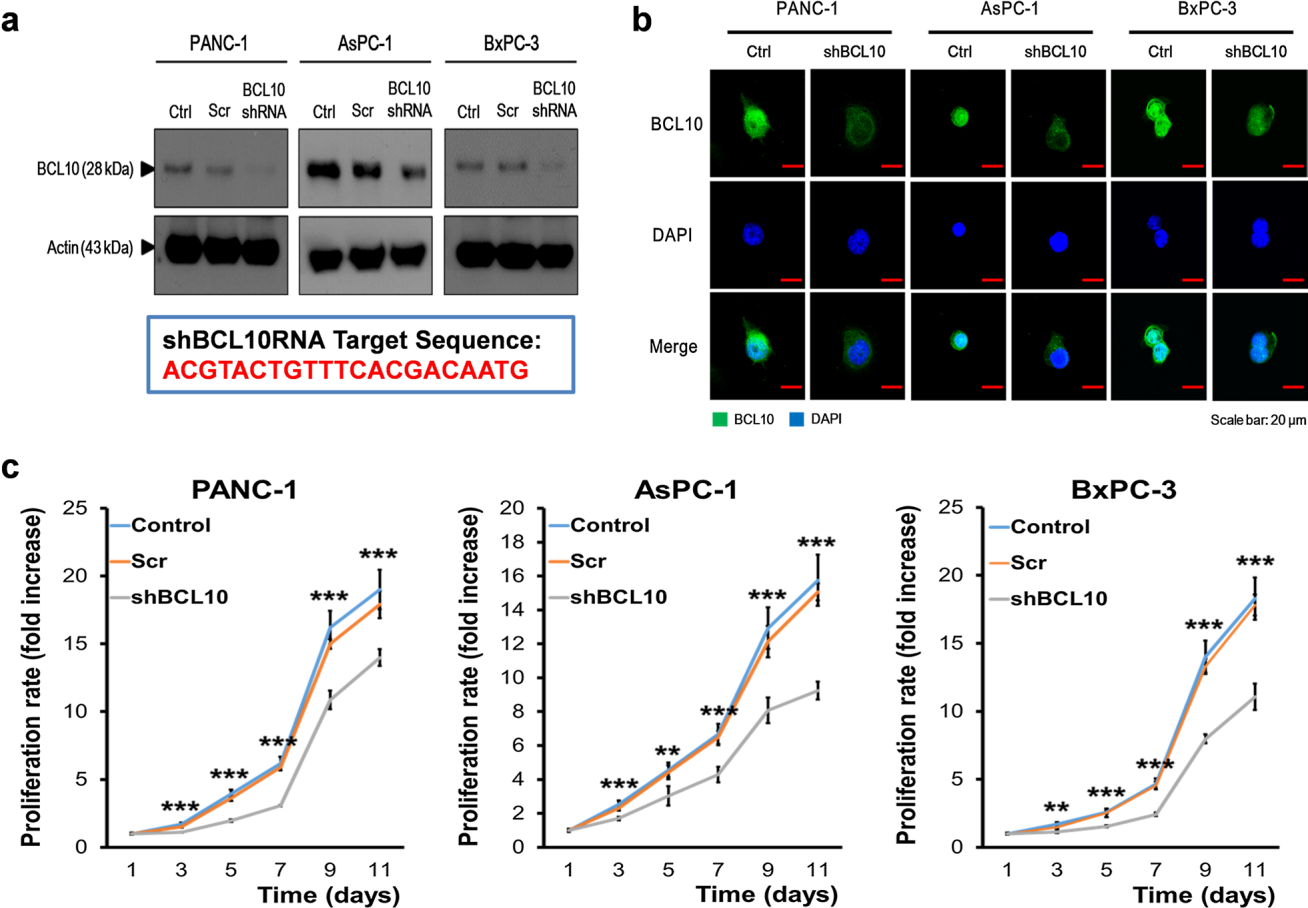
Silencing of BCL10 in the cells of PDAC using short hairpin RNA (shRNA). **a** The shRNA-expression lentiviral vector of BCL10 (Clone ID: TRCN0000359256), specifically targets C-terminal region of BCL10, including Ser^231^ phosphorylation site (ACGTACTGTTTCACGACAATG). Three PDAC cell lines (PANC-1, AsPC-1, and BxPC-3) were infected with pGIPZ lentivirus (scrambled, Scr) and BCL10 shRNA lentivirus. The protein expression of β-actin and BCL10 was examined by western blot analysis. **b** Transfection with BCL10 shRNA (shBCL10) significantly decreased the levels of BCL10 in nucleus of three PDAC cell lines. Cells were labeled with anti-BCL10 primary antibody and DyLight 488 conjugated secondary antibody. The distribution of BCL10 was observed by immunofluorescence using confocal microscopy. Nuclei were counterstained with DAPI. Scale bar: 20 μm. **c** shBCL10 transfected PDAC cells exhibited low proliferative ability. The control, scrambled, and shBCL10 transfected cells from PANC-1, AsPC-1, and BxPC-3 cell lines were plated at 1000 cells/well in 96-well plate respectively. Then, cell proliferation rate was evaluated using a Premixed WST-1 Cell Proliferation Reagent after every 2 days. **p < 0.01; ***p < 0.001. *PDAC* pancreatic ductal adenocarcinoma

To investigate the functions of BCL10 in PDAC cells, we first validated the localization of BCL10. The three PDAC cell lines were infected with BCL10 shRNA lentivirus, and then, assessed by western blot analysis. The data showed efficient knockdown of BCL10 after transfection with BCL10 shRNA (Fig. 1a). The immunofluorescence image from confocal microscopy showed that BCL10 was localized in nucleus and cytoplasm of all three PDAC cell lines (PANC-1, AsPC-1, and BxPC-3) (Fig. 1b). After transfection with BCL10 shRNA (shBCL10), the fluorescence intensity of BCL10 in cytoplasm and nucleus was markedly reduced in BxPC-3 cells, and BCL10 expression was mainly restricted to cytoplasm in PANC-1 and AsPC-1 cells (Fig. 1c).

We further examined whether the growth of PDAC cells was inhibited after BCL10 knockdown. As shown in Fig. 1c, transfection with shBCL10 significantly reduced cell proliferation in three PDAC cell lines. These results suggested that nuclear translocation of BCL10 may be a key event in the promotion of proliferation of these PDAC cells.

### Downregulation of BCL10 causes cell cycle arrest and enhances the cytotoxicities of chemotherapeutic agents in PDAC cells

**Fig. 2:**
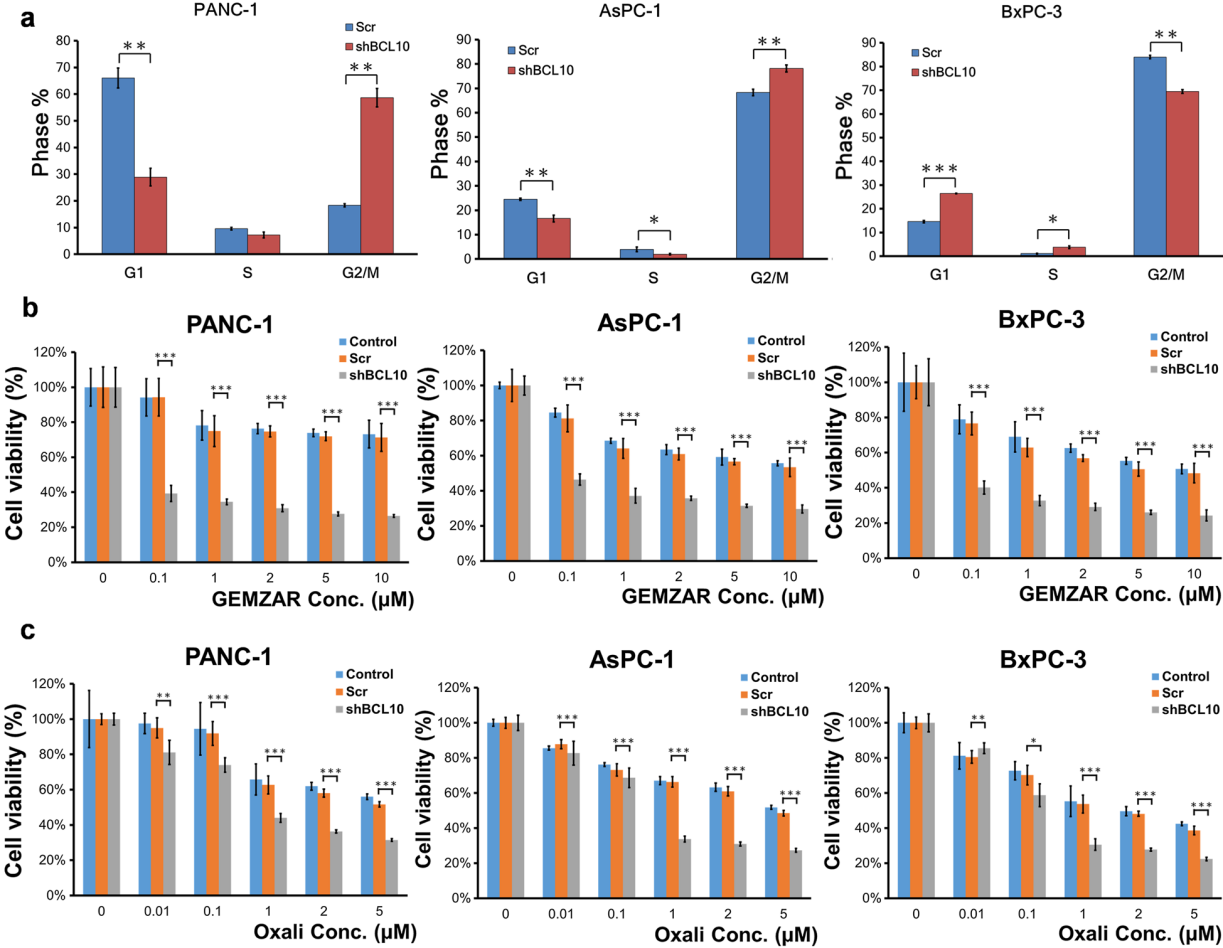
Inhibition of BCL10 causes differential cell cycle arrest and increases drug sensitivity in PDAC cell lines.** a** shBCL10 resulted in G2/M phase arrest in PANC-1 and AsPC-1 cells, but caused G1 phase arrest in BxPC-3. **b** The cell survival analysis showed that shBCL10 transfection significantly increased a dose-dependent inhibition of cell viability to gemcitabine (GEMZAR) treatment in PANC-1, AsPC-1, and BxPC-3 cells. **c** Similarly, shBCL10 transfection significantly increased a dose-dependent inhibition of cell viability to oxaliplatin (Oxali) treatment in PANC-1, AsPC-1, and BxPC-3 cells. The results are expressed for triplicates in each treatment group and measured each week. *p < 0.05; **p < 0.01; ***p < 0.001. *PDAC* pancreatic ductal adenocarcinoma

To investigate whether BCL10-mediated suppression of proliferation of pancreatic cancer cells is via the cell cycle arrest, we assessed the effects of shBCL10 transfection on cell cycle regulation in three PDAC cell lines. As shown in Fig. 2a, in *KRAS* mutant PDAC cells, PANC-1 and AsPC-1, there was a significant increase in the number of cells in the G2/M phase and significant decrease in the number of cells in the G1 phase. In the *KRAS* wild-type PDAC cell line, BxPC-3, we observed that there was a significant increase in the number of cells in the G1 phase and significant decrease in the number of cells in the G2/M phase (Fig. 2a). These findings indicated that knockdown of BCL10 differentially inhibited cell cycle progression in PDAC cell lines; G1 phase arrest was noted in wild-type *KRAS* cell lines, whereas G2/M phase arrest was noted in mutant *KRAS* cell lines.

We further evaluated whether PDAC cells are sensitive to conventional chemotherapy after silencing of BCL10 using shBCL10. The PDAC cell lines were treated with various doses of gemcitabine and oxaliplatin for 72 h, respectively. The cytotoxicity assay showed that BCL10-silenced cells exhibited significantly lower cell survival rate compared with scrambled cells after treatment with the same drug doses of gemcitabine (Fig. 2b) and oxaliplatin (Fig. 2c) in the three PDAC cell lines.

### Downregulation of BCL10 significantly decreases the expression of cell cycle-regulatory and NF-κB-related proteins, and inhibits NF-κB activation

It has previously been demonstrated that Cyclin D1 is involved in the G1 phase, while Cyclin E, and Cyclin A participate in the transition to the G1/S phase [32]. In contrast to G1/S phase, CDC2, CDC25C, and Cyclin B1 are involved in the G2/M phase [33]. The cyclin-dependent kinase inhibitors p21 and p27 regulate different stages of the cell cycle, including the G1 phase, and the transition of G1/S and G2/M phases by inhibiting the functions of the various CDK proteins as indicated [34–36]. In the regulation of G1 phase and G1/S transition, p21 and p27 preferentially inhibit the activation of the complex of CDK4/6/Cyclin D and CDK2/Cyclin E, and also restrains the activity of the interaction between CDK2 and Cyclin A, whereas in the regulation of G2/M phase progression, both p21 and p27 inhibit the activation of the CDK1/Cyclin B complex [34–36]; therefore, in this study, we assessed whether silencing BCL10 using shBCL10 can affect the cell cycle proteins that are involved in the G1 phase and G1/S transition, including Cyclin A, Cyclin D, Cyclin E, CDK2, CDK4, p21, and p27, and G2/M phase arrest, and in the transition of the G2/M phase, including CDC2, p-CDC2, CDC25C, p-CDC25C, Cyclin B1, p21, and p27, in BxPC3 and PANC-1 cells.

**Fig. 3:**
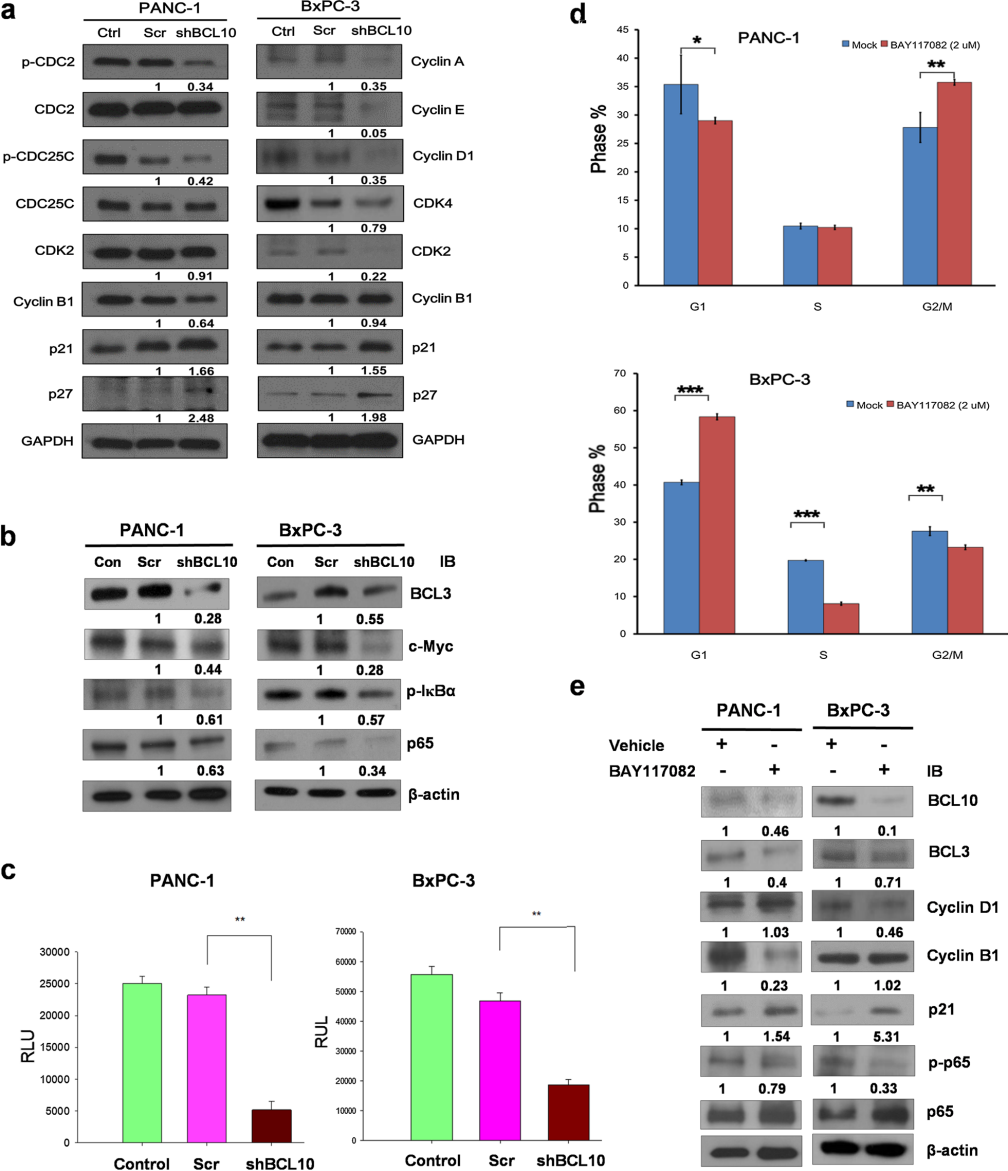
Inhibition of BCL10 downregulates cell cycle proteins expression and NF-κB activation in PDAC cell lines. **a** In total cell lysates, transfection with shBCL10 downregulated the expression of p-CDC2, p-CDC25C, Cyclin B1 in PANC-1 cell lines and the expression of Cyclin A, E, and D1, CDK2, and CDK4 in BxPC-3 compared to scrambled (Scr) group and control (Con) group. When compared to scrambled (Scr) group and control (Con) group, shBCL10 transfection upregulated the expression of p21 and p27 in both PANC-1 and BxPC-3 cells. **b** shBCL10 transfection decreased the expressions of BCL3, c-Myc, p-IκBα, and NF-κB (p65) compared to scrambled (Scr) group and control (Con) group in nuclear lysates of both PANC-1 and BxPC-3 cells. **c** The results from three independent experiments using shBCL10-transfected PANC-1 and BxPC-3 cells are presented as relative luciferase units (RLU) per milligram of protein (NF-κB-Luc promoter activity-luciferase assay) (**p < 0.01). **d** PANC-1 cells and BxPC-3 cells were treated with BAY117082 (2 µM) for 24 h and compared with vehicle (0.01% DMSO) treated groups. *p < 0.05; n = 3. **e** PANC-1 cells and BxPC-3 cells were treated as described in (**d**), the expressions of BCL10, BCL3, p-p65, and p65 in nuclear lysates of PANC-1 cells and BxPC-3 cells, and the expressions of Cyclin B1, Cyclin D1, and p21 in total lysates were determined by western blotting. β-actin was used as the loading control. *PDAC* pancreatic ductal adenocarcinoma

As shown in Fig. 3a, we found that in PANC-1 cells, shBCL10 transfection downregulated the expression of p-CDC2, p-CDC25C, and Cyclin B1, and upregulated the expression of p21 and p27, while the expression of CDK2 was not affected (Fig. 3a). In BxPC-3 (wild-type *KRAS*) cells, we revealed that shBCL10 downregulated the expression of Cyclin A, Cyclin D1, Cyclin E, CDK2, and CDK4, and upregulated the expression of p21 and p27, but did not affect the expression of Cyclin B (Fig. 3a). These results indicated that BCL10 inhibition can result in differential cell cycle arrest in the two different pancreatic cancer cell lines: mutant *KRAS* cell lines (G2/M arrest) and wild-type *KRAS* cell lines (G1 arrest).

As shown in Fig. 3b, shBCL10 transfection inhibited the expression of nuclear BCL3 and p-IκBα, both of which are recognized as important regulators of NF-κB in both PANC-1 and BxPC-3 cells. In addition, shBCL10 transfection downregulated nuclear expression of NF-κB (p65) and c-Myc in both PANC-1 and BxPC-3 cells. As indicated by the results from the NF-κB-Luc promoter activity assay, the transcription of NF-κB-dependent genes, led by the nuclear translocation and DNA binding of NF-κB, was downregulated in both PANC-1 and BxPC-3 cells after transfection with shBCL10 (Fig. 3c).

To confirm the role of BCL10-mediated activation of NF-κB in cell cycle regulation, we treated PANC-1 and BxPC-3 cells with an NF-κB inhibitor, BAY117082 (2 µM), for 24 h; cell cycle distribution analysis, as shown in Fig. 3d, revealed that the inhibition of NF-κB did cause cell cycle arrest at G2/M in PANC-1 cells and at G1 arrest in BxPC-3 cells. As shown in Fig. 3e, we showed that expression of Cyclin B1 (PANC-1), Cyclin D1 (BxPC-3), and nuclear BCL10, BCL3, c-Myc, and p-p65 (both BxPC-3 and PANC-1) was downregulated after BAY117082 (an NF-κB inhibitor) treatment, while p21 was upregulated after BAY117082 treatment. However, BAY117082 did not affect the expression of Cyclin D1 in PANC-1 and the expression of Cyclin B1 in BxPC-3 cells. We concluded that BCL10, at least in part, regulates the growth and the cell cycles progression of PDAC cells via NF-κB-dependent signaling.

### Inhibition of BCL10 expression reduces tumor growth of PDAC in a xenograft model

**Fig. 4:**
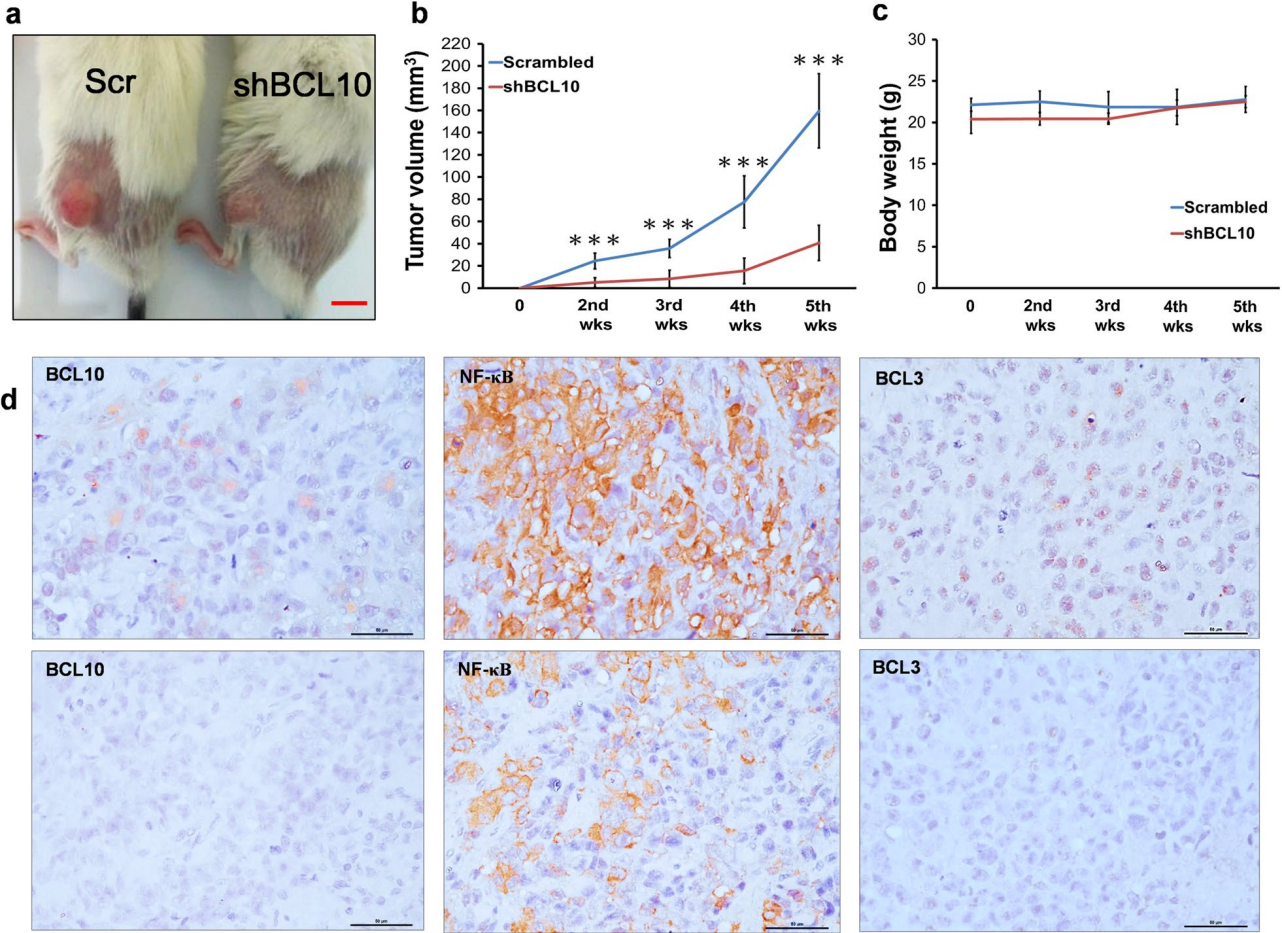
Silencing of BCL10 significantly inhibited tumor growth of PDAC in a xenograft model. **a** Approximately 1 × 10^6^ scrambled (Scr) PANC-1 cells and shBCL10-treated PANC-1 cells were implanted subcutaneously into both flanks of NOD/SCI D mice. Representative image shows that the xenograft tumor size was suppressed in shBCL10-treated group. Mice were sacrificed 6 weeks after implantation. Scale bar: 10 mm. **b** The shBCL10-treated group resulted in 78% reduction of xenograft tumor volume compared with scrambled group. The results of tumor volume are expressed as n = 6 in each treatment group and measured each week. ***p < 0.001, compared to scrambled group. **c** There is almost no difference in body weight between scrambled group and shBCL10-transfected group. **d** The expression patterns of BCL10, BCL3, and NF-κB in tumor samples of PANC-1-xenograft model (upper panel, scrambled group; lower panel, shBCL10-treated group). *PDAC* pancreatic ductal adenocarcinoma

To assess the anti-tumor effect of BCL10 knockdown in PDAC xenograft model, PANC-1 cells treated with or without shBCL10 transfection were inoculated into the flanks of mice. Tumor volume was recorded from appearance of initial tumor burden (Fig. 4a and b). When compared with scrambled group of PANC-1 xenograft tumors, we observed that the tumor growth was significantly inhibited after transfection with shBCL10 (Fig. 4a). The shBCL10-transfected group exhibited 78% reduction of tumor volume compared with scrambled group at 5 weeks (Fig. 4b). There were no significant differences in the body weight between scrambled PANC-1 group and shBCL10-transfected PANC-1 group (Fig. 4c). The nuclear expression levels of BCL10, BCL3, and NF-κB (p65) in tumor cells were also downregulated in shBCL10-transfected PANC-1 group when compared with scrambled PANC-1 group (Fig. 4d).

### Nuclear expression of BCL10 in clinical sample is closely associated with the poor overall survival of patients with recurrent, advanced, and metastatic PDAC

The patients’ clinical characteristics are summarized in Table 1. The median age for all the patients was 59 years old (range: 27–82) and all patients had histologically confirmed PDAC. Approximately one third of the patients (49 of 136 cases, 36%) had a history of smoking tobacco. Of 136 patients, 80 patients (58.8%) were initially diagnosed with stage IV pancreatic cancer, and the most common metastatic sites were liver, whereas 19 patients had locally advanced pancreatic cancer. Of 136 patients, 37 patients had local recurrence (n = 15) and/or distant metastasis (n = 22) after undergoing surgery with curative intent. After a median follow-up of 24.8 months [95% confidence interval (CI) 13.2–36.4 months], the median OS after commencing chemotherapy for locally advanced and metastatic PDAC or developing recurrence and/or distant metastasis of the entire group was 7.54 months (95% CI 6.11–8.98 months).

**Table 1:** Clinicopathologic features between nuclear BCL10-negative and nuclear BCL10-positive groups of patients with PDAC

	Nuclear BCL10 expression			
	Total (N)	Negative	Positive	p-value
Number	136	78 (57.4%)	58 (42.6%)	
Age				0.837^†^
Median	59.0	58.0	61.0	
Range	27–82	35–82	27–76	
Sex				0.102^‡^
Men	83 (61.0%)	43 (55.1%)	40 (69.0%)	
Women	53 (39.0%)	35 (44.9%)	18 (31.0%)	
Smoking				0.262^‡^
No	87 (64.0%)	53 (67.9%)	34 (58.6%)	
Yes	49 (36/0%)	25 (32.1%)	24 (41.4%)	
Alcohol				0.937^‡^
No	98 (72.1%)	56 (71.8%)	42 (72.4%)	
Yes	38 (27.9%)	22 (28.2%)	16 (27.6%)	
Stage				0.743^§^
Recurrent	37 (27.2%)	23 (29.5%)	14 (24.1%)	
Stage IIIB	19 (14.0%)	9 (11.5%)	10 (17.2%)	
Stage IV	80 (58.8%)	46 (59.0%)	34 (58.6%)	
Metastases				0.667^§^
Peritoneal	12 (8.8%)	7 (9.0%)	5 (9.6%)	
Liver	83 (61.0%)	47 (60.2%)	36 (62.1%)	
Other sites^a^	23 (16.9%)	12 (15.4%)	11 (19.0%)	
Regional LNs	18 (13.2%)	12 (15.4%)	6 (10.3%)	
Chemotherapy				0.306^‡^
Gem-alone	44 (32.4%)	28 (35.9%)	16 (27.6%)	
Gem-based	92 (67.6%)	50 (61.1%)	42 (72.4%)	
Response				0.423^§^
CR + PR	11 (8.1%)	6 (7.7%)	5 (8.6%)	
SD + PD	85 (62.5%)	51 (65.4%)	34 (58.6%)	
NA	40 (29.4%)	21 (26.9%)	19 (32.8%)	
NF-κB expression^b^				< 0.001^‡^
Negative	58 (61.1%)	49 (84.5%)	9 (24.3%)	
Positive	37 (38.9%)	9 (15.5%)	28 (75.7%)	

*PDAC* pancreatic ductal adenocarcinoma, *N* number, *LNs* lymph nodes, *Gem* gemcitabine, *CR* complete remission, *PR* partial remission, *SD* stable disease, *PD* progressive disease, *NA* non-analyses or difficult to assess the response

^a^Other sites: included extra-liver lesions, such as lung, adrenal gland metastases

^b^NF-κB expression was examined in tumor cells of selected 95 cases of PDAC

^†^P values (2-sided) were calculated using the Student *t* test

^‡^P values (2-sided) were calculated using the Χ^2^ test or the Fisher exact test

^§^P values (2-sided) were calculated using 1-way analysis of variance

**Fig. 5:**
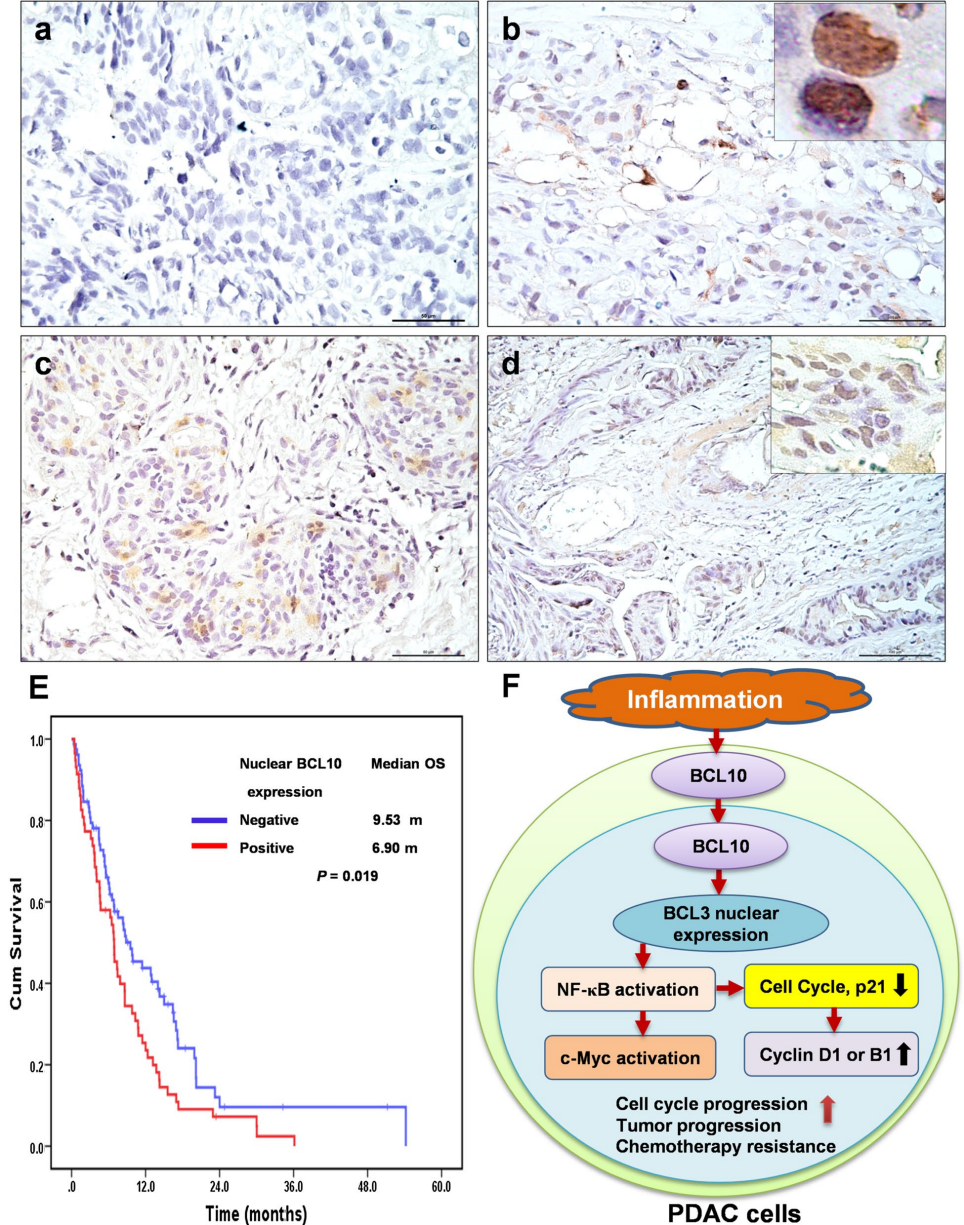
Expression of nuclear BCL10 in tumor cells and its association with survival of patients with recurrent, advanced and metastatic PDAC. **a** Representative images of negative BCL10 expression in tumor specimens of PDAC. **b** Representative images of nuclear BCL10 expression in biopsy tumor specimens of PDAC. Right upper inset (nuclear expression of BCL10, 100X magnification). **c** Representative images of moderate nuclear BCL10 expression in tumor specimens of PDAC. **d** Representative images of strong nuclear BCL10 expression in tumor specimens of PDAC. Right upper inset (nuclear expression of BCL10, 40X magnification). **e** The Kaplan–Meier overall survival (OS) for all patients associated with the expression of nuclear BCL10. **f** Inflammation-related signals in the tumor microenvironment of PDAC upregulates BCL10 expression and induces BCL10 nuclear translocation; BCL10 nuclear expression results in BCL3 nuclear translocation and the activation of NF-κB. The activation of NF-κB further triggers its regulating transcriptional factors, including c-Myc and cell cycle proteins, such as Cyclin D1 in wild-type *KRAS* PDAC, and Cyclin B1 in *KRAS* mutant PDAC. These signaling pathways further promote cell cycle progression, tumor progression, and contribute to chemotherapy resistance of PDAC. Inhibition of nuclear BCL10 translocation attenuates the activation of NF-κB, leading to the decreased expression of its regulatory cell cycle proteins, Cyclin D1 (wild-type *KRAS*) or Cyclin B1 (mutant *KRAS*), and the altered expression of p21, and thus fosters G1 arrest and G_2_/M arrest, respectively, and also enhances chemosensitivity and prolongs the survival of patients with PDAC. *PDAC* pancreatic ductal adenocarcinoma

The nuclear expression of BCL10 was detected in tumor cells of 58 patients (42.6%) (Fig. 5a–d). The difference in the demography, including age, sex, smoking history, and alcoholism were not significant between patients with nuclear BCL10 expression in tumors and those without nuclear BCL10 expression in tumors (Table 1). Of 136 patients, 96 patients (70.6%) with available response to gemcitabine-based chemotherapy; however, patients without nuclear BCL10 expression did not exhibit better response (complete and partial remission) to gemcitabine-based chemotherapy than those with nuclear BCL10 expression [5/39 (12.8%) versus 6/57 (10.5%), p = 0.753]. Patients with nuclear BCL10 expression had significantly worse prognoses than those without nuclear BCL10 expression [median OS after starting chemotherapy: 6.90 months (95% CI 5.77–8.03) versus 9.53 months (95% CI 5.89–13.18), p = 0.019]. The 1-year OS rates for patients with nuclear BCL10 expression and for patients without nuclear BCL10 expression were 23.6% (95% CI 12.4–34.8%) and 43.7% (95% CI 32.1–55.3%), respectively (Fig. 5d).

Among 95 patients with available tissues to assess the expression of NF-κB (p65), we found that the nuclear BCL10 expression was significantly associated with nuclear NF-κB expression (p < 0.001, Table 1). Similarly, patients with nuclear NF-κB expression (n = 37) had a shorter OS than those without nuclear NF-κB expression (n = 58) [median survival: 6.50 months (95% CI 3.22–9.78) versus 9.90 months (95% CI 3.96–15.84), p = 0.025]. The 1-year OS rates for patients with nuclear NF-κB expression and for patients without nuclear NF-κB expression were 31.9% (95% CI 16.2–47.6%) and 46.5% (95% CI 32.6–53.6%), respectively.

## Discussion

In the current study, we demonstrated that inhibition of BCL10 using shBCL10 significantly inhibited the cell proliferation of the three PDAC cell lines and the tumor growth of mice bearing PANC-1 xenograft tumors. The possible mechanisms of attenuation of cell proliferation of PDAC cells via downregulation of BCL10 are through arresting of cell cycle and inactivation of NF-κB-signaling. The biological significance of nuclear BCL10 expression was further validated in tumor cells of patients with advanced, metastatic, and recurrent and/or metastatic PDAC after curative surgery, in which the nuclear BCL10 expression significantly correlated with nuclear NF-κB expression and poor OS (after commencing chemotherapy) of these patients.

In contrast to hematological malignancies, such as MALT lymphoma [15, 18, 19], BCL10 was rarely found in the nucleus of solid tumors [21, 22]. In this study, BCL10 was expressed in cytoplasm as well as in nucleus of all three PDAC cell lines. After removal of Ser^231^ of BCL10, most of the BCL10 was localized in the cytoplasm of these PDAC cell lines. In addition, nuclear expression of BCL3 was downregulated after shBCL10 treatment. Furthermore, inhibition of BCL10 by shBCL10 transfection downregulates phosphorylation of IκBα and NF-κB (p65), and attenuates NF-κB activation and its regulated protein, c-Myc. In another study of natural killer T cell lymphoma, Chan et al. demonstrated that interleukin 2 triggered NF-κB activation via PI3K/AKT/BCL10/BCL3 signaling pathway, and that nuclear BCL10 expression was closely associated with NF-κB activation [37]. Ismail et al. also reported that in breast cancer cell line, T47D, nuclear BCL10 was subcellularly localized into the cytoplasm after transfection with siBCL10 [38]. These findings indicated that BCL10 may behave as a shuttle between nucleus and cytoplasm in certain cancer cells, and the shuttling of BCL10 may be dependent on the inflammation-related NF-κB signaling and nuclear BCL3-associated export.

In pancreatic cancers, mutational activation of *KRAS* or alterations in PI3K regulation caused by *phosphatase and tensin homolog* (*PTEN*) loss can enhance NF-κB activation and NF-κB-associated activation of downstream cytokine genes (including c-Myc and Cyclin D1) [39–43], and thus, contribute to progression of these tumors. These findings were in corroboration of current study showing the vital role of nuclear BCL10 in mediating activation of the NF-κB and its downstream gene, c-Myc, and in contributing to poor prognosis.

In this study, we also assessed whether nuclear BCL10 expression correlated with *KRAS* mutation in patients with locally advanced and metastatic PDAC. We revealed that among 75 cases of PDAC, *KRAS* mutations in codon 12 (exon 2) were detected in 63 (84%) patients, and nuclear BCL10 expression was not associated with the *KRAS* mutation [*KRAS* mutant cases: positive nuclear BCL10 expression rate was found to be 49.2% (31/63) versus *KRAS* wild-type case: positive nuclear BCL10 expression rate was found to be 33.3% (4/12), p = 0.302] (Additional file 2: Table S1). These findings indicate that nuclear BCL10 expression may participate in the molecular pathogenesis of PDAC in both mutant *KRAS* and wild-type *KRAS* PDAC cells.

In this study, we demonstrated that BCL10 inhibition can result in differential cell cycle arrest in the two different pancreatic cancer cell lines: mutant *KRAS* cell lines (G2/M arrest) and wild-type *KRAS* cell lines (G1 arrest). The possible mechanisms why the inhibition of BCL10 can contribute to different cell cycle arrests in mutant *KRAS* and wild-type *KRAS* PDAC cell lines may be resulted from the following clues. In contrast to wild-type *KRAS* PDAC, activation of PI3K/AKT/mTOR signaling was predominant in *KRAS* mutant PDAC [30, 31]. Previous studies have suggested that persistent activation of PI3K/AKT/mTOR signaling may cause the progression of the G2/M transition in PDAC cells [44, 45]. In 7,12-dimethylbenzanthracene (DMBA)-implantation-induced pancreatic cancer rats, in which the oncogenesis of pancreatic cancer (PC) is mainly triggered by the *KRAS* mutation-related signaling pathway [46], Guo et al. revealed that Cyclin B1 was upregulated in the DMBA-treated group when compared with the NaCl crystal-treated group through oligonucleotide microarray approaches [47]. In an analysis of the association between Cyclin B1 expression and the prognosis of 241 patients with PC, Zhou et al. showed that patients with tumors expressing high levels of Cyclin B1 (grade 2, > 30% positive immunostaining) had a poorer median overall survival than those with low-level expression of Cyclin B1 (12.4 months versus 22.8 months, p = 0.01) [48]. In human samples of PC, Ito et al. showed that cell division cycle (CDC)2 expression was significantly higher in pancreatic adenocarcinoma than in intraductal or cystic adenocarcinoma [31/50 (54.8%) versus 3/13 (23.1%), p = 0.0418], and the overexpression of CDC2 was also demonstrated to be closely associated with lymph node metastasis and a high Ki-67 labeling index in these patients with PC [49]. Our current findings showed that inhibition of BCL10 downregulated the expression of Cyclin B1, p-CDC2, and p-CDC25C in the *KRAS* mutant PDAC cell line, PANC-1, indicating that BCL10 may be involved in the regulation of important checkpoints that regulate cell cycle progression at the G2/M transition in *KRAS* mutant PDAC.

It was noted that knockdown of BCL10 by shBCL10 in the wild-type *KRAS* cell line, BxPC-3, resulted in the decreased expression of cyclin-dependent kinases (CDKs) and cyclins that control the process of G1 phase and G1/S transition, such as Cyclin D1, Cyclin E, CDK2, CDK4, and Cyclin A. We also found that the inhibition of NF-κB by BAY117082 caused cell cycle arrest in G1, downregulation of Cyclin D1 expression, and upregulation of p21 expression in BxPC-3 cells but did not affect Cyclin B1, indicating that BCL10 is involved in the activation of NF-κB and its regulated cell cycle protein, Cyclin D1, in wild-type *KRAS* PDAC. These results support our previous finding that inhibition of BCL10 with BCL10 small interfering RNA caused G1 cell cycle arrest in four human cervical cancer cell lines, C33A, HeLa, SiHa, and CaSki, in which all were wild-type *KRAS*, and silencing BCL10 in SiHa cells significantly downregulated Cyclin D1 expression and the activation of NF-κB [21]. Previous studies have also demonstrated that Cyclin D1 overexpression is closely associated with the poor prognosis in post-operative pancreatic cancer patients [50, 51]. Further exploration of the association between Cyclin D1 expression and the nuclear expression pattern of BCL10 in patients with wild-type *KRAS* PDAC is required.

In addition to wild-type *KRAS* PDAC cell lines, we revealed that BAY11708, an NF-κB inhibitor, downregulated Cyclin B1, upregulated p21, and caused G2/M phase arrest, but did not affect Cyclin D1 in *KRAS* mutant PDAC cell lines (PANC-1). Using an in vitro BRL-3A rat liver cell model, Zhang et al. showed that Cyclin B1, a downstream molecule of NF-κB, was stimulated through activation of recombinant rat IL-18-triggering NF-κB signaling [52]. Further investigation of the association between NF-κB and Cyclin B1 and the biological functions of NF-κB/Cyclin B1 signaling in *KRAS* mutant PDAC are warranted.

In addition to the diverse suppression of CDKs and cyclins in different mutant *KRAS* and wild-type *KRAS* PDAC cell lines, we found that inhibition of BCL10 upregulated the expression of p21 and p27 in both mutant *KRAS* and wide-type *KRAS* PDAC cell lines, suggesting that BCL10 may participate in the regulation of or affect the function of p21 and p27, both regulating cell cycle progression at the G1/S and G2/M boundary, and thus contribute to the progression and drug resistance of PDAC. In an in vitro cell line study, Yadav et al. showed that gatifloxacin enhances the cytotoxicities of gemcitabine in two *KRAS* mutant PDAC cell lines, MIA PaCa-2 and PANC-1, through upregulation of p21 and p27 expression and subsequent blocking of G1/S phase progression [53]. A previous study showed that p21 was significantly associated with NF-κB activation and was involved in the regulation of the G2/M phase cell cycle in T-cell leukemic cell lines [54]. Our findings revealed that inhibition of NF-κB by BAY117082 upregulated the expression of p21 in both PDAC cell lines, supporting our findings that inhibition of BCL10 attenuated NF-κB activation and further increased p21 expression, thus blocking cell cycle progression in the G1/S phase (BxPC-3) and G2/M phase (PANC-1), respectively. Taken together, our results suggest that BCL10 may be involved in the activation of NF-κB signaling and its regulated cell cycle proteins and the alteration of p21 and p27, thus controlling different cell cycle transitions and further promoting cell proliferation and progression in different types of *KRAS* in PDAC cells (Fig. 5f).

The association between nuclear BCL10 expression and patients’ prognosis has been demonstrated in a variety of hematologic malignancies, including our and other investigators’ studies showing *Helicobacter pylori*-independence (lack of tumor regression after antibiotics treatment) of gastric lymphoma [18, 19, 55–57], the short failure-free survival in ocular adnexal MALT lymphoma [58], and locally aggressive tumors in cutaneous MALT lymphoma [59]. Holzmann et al. reported that higher mRNA expression of BCL10 was detected in eight pancreatic cancer cell lines and in all analyzed pancreatic cancer tissues, including primary tumors and surgical margins, indicating a role of BCL10 in the molecular pathogenesis of pancreatic cancer [60].

Previous studies have demonstrated that activation of NF-κB results in significant resistance of pancreatic cancer cells to chemotherapeutic agents, including gemcitabine and oxaliplatin [24, 61]. Our in vitro study showed that inhibition of BCL10, using shBCL10, might enhance cytotoxicities of gemcitabine and oxaliplatin. Furthermore, we revealed the significant correlation between nuclear expression of BCL10 and poor OS in locally advanced and metastatic pancreatic cancer patients receiving gemcitabine-based chemotherapy. These findings indicated that BCL10 may be a potential target for treating locally advanced or metastatic pancreatic cancer.

## Conclusions

In the present study, we found that BCL10 residing in the cytoplasm and nucleus of PDAC cells relays inflammation-mediated signals to activate NF-κB, and that nuclear BCL10 expression is associated with poor prognosis of patients with locally advanced and metastatic PDAC. These findings suggested that nuclear BCL10 may act as an important factor for promoting cell proliferations and chemoresistance, and thus, for increasing aggressiveness of PDAC cells. Further study of the biological significance of nuclear BCL10 in mediating NF-κB activation and in differentially regulating cell cycle proteins in PDAC cells can help us in developing novel BCL10-related therapeutic strategies for this subgroup of tumors.

## Supplementary Information

**Additional file 1: Figure S1.** The mRNA and amino acid sequences of BCL10.

**Additional file 2: Table S1.** The association between K-RAS mutation status and the expression pattern of nuclear BCL10 in 75 patients of pancreatic ductal adenocarcinoma.

## Data Availability

The datasets used and/or analyzed in the current study are available from the corresponding author on reasonable request.

## Abbreviations

PDAC: Pancreatic ductal adenocarcinoma
Shh: Sonic hedgehog (Shh)
NF-κB: Nuclear factor-κappa B
OS: Overall survival
BCL10: B-cell CLL/lymphoma 10
TNF-α: Tumor necrosis factor-alpha
ATCC: American Type Culture Collection (ATCC)
RPMI: Roswell Park Memorial Institute
shRNA: Short hairpin RNA
shBCL10: BCL10 shRNA transfected
SE: Standard error
Scr: Scrambled
Con: Control
PBS: Phosphate buffered saline
PI: Propidium iodide
CDK: Cyclin-dependent kinases
CDC: Cell division cycle
SDS: Sodium dodecyl sulfate
PAGE: Polyacrylamide gel electrophoresis
PVDF: Polyvinylidene difluoride
p: Phospho
IACUC: Institutional Animal Care and Use Committee
CI: Confidence interval
*PTEN*: Phosphatase and tensin homolog
RLU: Relative luciferase units
GEMZAR: Gemcitabine
Oxali: Oxaliplatin
