# Supplementary material for: Contribution of nuclear BCL10 expression to tumor progression and poor prognosis of advanced and/or metastatic pancreatic ductal adenocarcinoma by activating NF-κB-related signaling
Source: Cancer Cell Int. 2021 Aug 19;21:436. doi: 10.1186/s12935-021-02143-z (PMC8375138; doi:10.1186/s12935-021-02143-z)
Supplement: Supplementary file 1 — Additional file 1: Figure S1. The mRNA and amino acid sequences of BCL10. [file 12935_2021_2143_MOESM1_ESM.pdf]

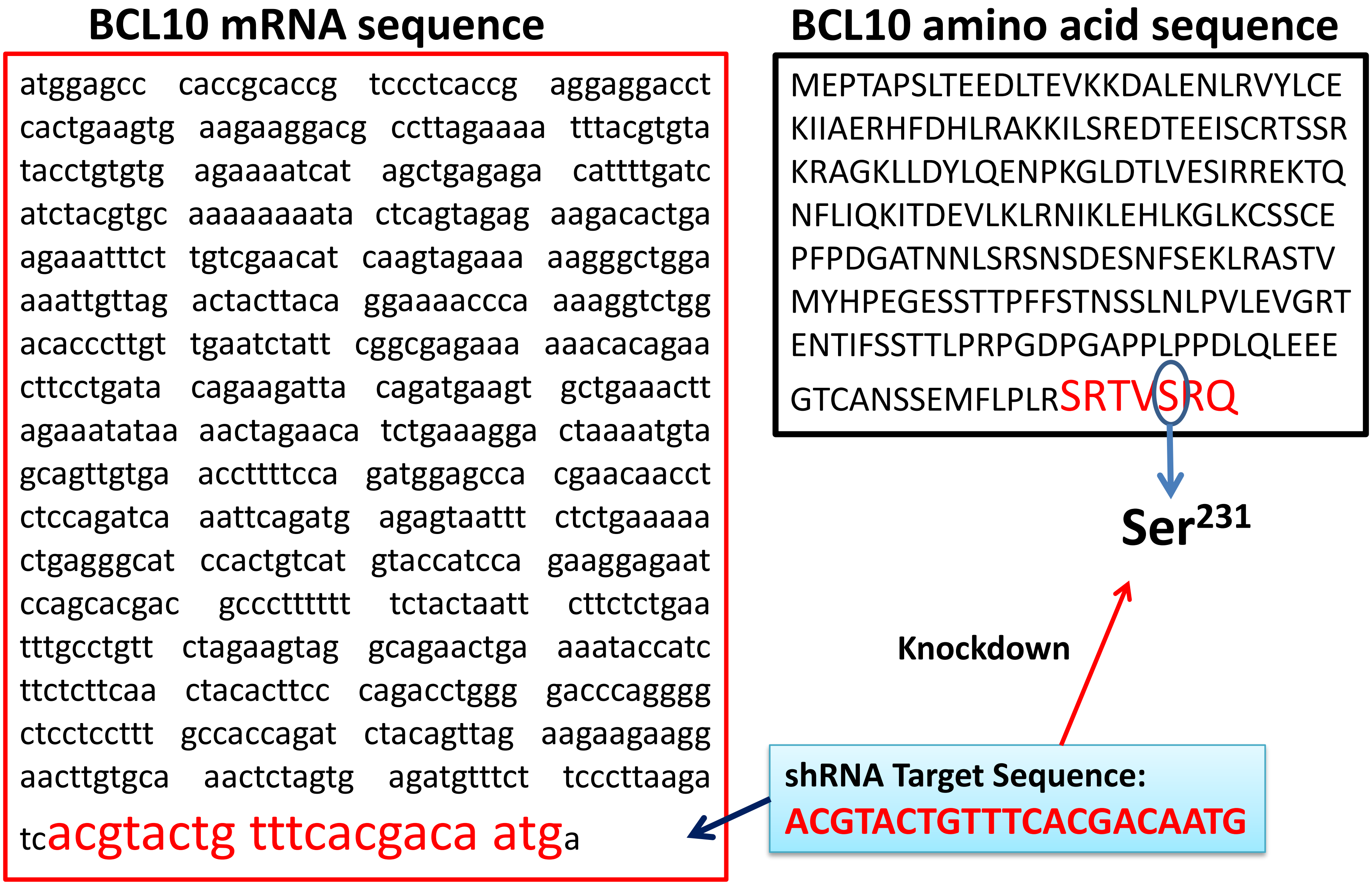

Supplementary Figure 1
