# Supplementary material for: Contribution of nuclear BCL10 expression to tumor progression and poor prognosis of advanced and/or metastatic pancreatic ductal adenocarcinoma by activating NF-κB-related signaling
Source: Cancer Cell Int. 2021 Aug 19;21:436. doi: 10.1186/s12935-021-02143-z (PMC8375138; doi:10.1186/s12935-021-02143-z)
Supplement: Supplementary file 2 — Additional file 2: Table S1. The association between K-RAS mutation status and the expression pattern of nuclear BCL10 in 75 patients of pancreatic ductal adenocarcinoma. [file 12935_2021_2143_MOESM2_ESM.docx]

**Additional file 2: Table S1. The association between *KRAS* mutation status and the expression pattern of nuclear BCL10 in 75 patients of pancreatic ductal adenocarcinoma**

| *KRAS* |  | Nuclear BCL10 expression | |  |
| --- | --- | --- | --- | --- |
| **Codon 12 mutation** | **Total (N)** | **Negative** | **Positive** | **p-value** |
| **Number** | 75 (100%) | 40 (53.3%) | 35 (46.7%) |  |
|  |  |  |  | 0.557† |
| **Wilde type** | 12 (16.0%) | 8 (20.0%) | 4 (11.4%) |  |
| **GAT mutation** | 40 (53.3%) | 19 (47.5%) | 21 (60.0%) |  |
| **GTT mutation** | 8 (10.7%) | 6 (15.0%) | 2 (5.7%) |  |
| **CGT mutation** | 3 (4.0%) | 2 (5.0%) | 1 (2.9%) |  |
| **Double mutation** | 12 (16.0%) | 5 (12.5%) | 7 (20.0%) |  |

† p values (2-sided) were calculated using 1-way analysis of variance.
